# Time to complete contemporary dental procedures – estimates from a cross-sectional survey of the dental team

**DOI:** 10.1186/s12903-023-03671-y

**Published:** 2023-11-25

**Authors:** Christian Bannister, Anwen Louise Cope, Anup Karki, Paul Harper, Sarah Peddle, Brenda Walters, Michael Allen, Ivor Gordon Chestnutt

**Affiliations:** 1 Dental Public Health, Cardiff University School of Dentistry, Cardiff, UK; 2 School of Mathematics, Cardiff University, Cardiff, UK; 3 Public Health Wales, Cardiff, UK; 4 Patient and Public Representative, Cardiff, UK; 5 Cardiff and Vale University Health Board, Cardiff, UK; 6 Cardiff University Dental Hospital and School, Heath Park, Cardiff, CF14 4XY UK

**Keywords:** Dentistry, Service planning, Time, Survey, Workforce.

## Abstract

**Background:**

There are few contemporary studies on the time taken to complete dental procedures, those most heavily relied on in the United Kingdom date back to 1999.

**Objectives:**

This work aimed to establish how long members of the dental team took to complete specific dental procedures, relevant to their scope of practice.

**Methods:**

Data were collected via a purposive sample of 96 dentists, dental hygienists/therapists and dental nurses. Via an online survey, participants were asked to state the mean, minimum and maximum time they estimated that they took to complete individual dental procedures.

**Results:**

The mean time taken to complete procedures common to both dentists and dental hygienists/therapists ranged from 3.7 to 4 min respectively for clinical note reading prior to seeing patients to 30.1 and 28 min to undertake root surface debridement. There were no significant differences between the time taken by dentists and dental hygienists/therapists to treat adult patients. However, in all but one procedure, dental hygienists/therapists reported taking longer (*p* = 0.04) to treat child patients.

**Conclusions:**

The data provided here represent an up to date assessment of the time taken to complete specific tasks by different members of the dental team. These data will be of value to service planners and commissioners interested in evolving a dental care system that employs a greater degree of skill-mix and preventively oriented care.

**Supplementary Information:**

The online version contains supplementary material available at 10.1186/s12903-023-03671-y.

## Introduction

Crucial to the effective and efficient delivery of healthcare is the time taken to undertake a given procedure. Time is as they say, money. Primary dental care involves the delivery of a large range of distinct procedures and knowledge of the time taken per procedure is important in determining the economics of making a dental practice function. There have been relatively few studies undertaken in the United Kingdom into the time taken to deliver dental care [1, 2]. The most commonly cited study is work undertaken by the British Dental Association known as “The 1999 Heathrow timings inquiry” [1]. This work took the form of a consensus exercise undertaken by 21 general dental practitioners.

However, since this work in the late 1990s there have been many developments in the delivery of primary dental care. These include changes in patient complexity – improved oral health in younger people, is counterbalanced by an ageing population with more complex oral care needs both in terms of the dental care required and presenting co-morbidities [3–5]. Whilst there have been efforts to produce contemporaneous timing of common dental procedures, these have had small sample sizes or only collected data about a limited number of procedures [2, 6].

In England and Wales, a major objective of reforms to the state-funded dental system (National Health Service [NHS]) is to transition the delivery of dental care from a system focused on restoration to one whose focus is on prevention [7–9]. Scotland and Northern Ireland have retained to a greater extent the fee-per-item system of funding that was abandoned in England and Wales in 2006. It is also the case that a desire to see greater use of skill-mix and a team where dentists undertake higher-skill procedures (e.g., crowns and bridges) and more routine procedures (e.g., restorations, fissure sealants, oral hygiene instruction) and are delegated to dental therapists, dental hygienists and dental nurses, is a long standing goal of system reform [10–15].

It is therefore timely to provide up-to-date data timings in contemporary dental practice.

## Aims and objectives

The study was designed to investigate, as estimated by dental professionals, the time taken to undertake a range of common, contemporary procedures in general dental practice. A comparison with previous estimates was also undertaken.

The context of the work was NHS general dental practice in Wales and the specific objective was to measure the estimated range and central tendency of the time taken by dentists, dental therapists, dental hygienists and dental nurses to carry out commonly performed dental procedures.

## Materials and methods

### Data collection

Data were collected by an online questionnaire (onlinesurveys.ac.uk). A common landing page was presented to all study participants which posed questions on role (dentist, dental hygienist/dental therapist, dental nurse, time since qualification, whether their primary dental qualification was awarded in the United Kingdom (UK) or from a non-UK institution, and whether they were registered as a specialist with the General Dental Council (the regulatory body for dental professionals in the UK).

Participants were also asked whether they worked in NHS general dental practice and what proportion of their time was devoted to NHS (as opposed to private) practice. Participants who indicated that they did not work in NHS general dental practice were thanked for their time and excluded from any further participation in data collection. Those who were engaged in NHS general dental practice were then, using skip logic, dependant on their role (dentist, hygienist/therapist, nurse), directed to a series of questions in which they were required to estimate the mean time, minimum and maximum time required to undertake procedures on 95% of occasions. Participants were requested to assume that they carried out all the procedures themselves, and to calculate the average timings by including the time required for the patient to enter the surgery, administration of local anaesthetic, performing the task, and patient departure from the surgery. A task was defined as one treatment item regardless of whether it was performed in one or more appointments.

The list of procedures was derived from the NHS Business services Authority General Clinical Data Set [16] and informed by the clinical experience of the investigators and were divided into procedures involving children and those involving adults.

The questionnaire was administered between 0ctober 2020 to January 2021. Participants were specifically asked to provide their estimates based on their experience prior to the COVID-19 pandemic.

Questions on time taken for inhalation sedation and IV sedation were answered by just a few respondents and these data were removed from the analysis.

### Study population

The study sample frame comprised all dental professionals working in Wales whose details were included on a database held by Health Education and Improvement Wales (HEIW). This organisation is responsible for the delivery of postgraduate education and training for continuous professional development for all dental professionals in Wales and the majority of dentists, dental hygienists, dental therapists and dental nurses in the country are registered with HEIW. An invitation was sent to all on the database with a covering letter explaining the purpose of the work and a link to the online survey. A reminder email was sent after 2 weeks.

### Data analysis

Descriptive analysis of the timings of common dental activities from the questionnaires (mean; range; % non-response) were performed separately for child and adult patients, for dentists, dental therapists/dental hygienists, and dental nurses, and compared. Wilcoxon signed rank tests were used to determine the significance of differences between estimated times for undertaking between dentists and hygienists/therapists. Analyses were undertaken in SPSS (IBM SPSS Statistics v27). Significance level was set at 0.05.

### Sample size determination

The sample size calculation was based on estimating the length of an adult dental examination, the most commonly performed dental procedure in NHS Wales. In the 1999 BDA Heathrow Timing Inquiry the mean length of a dental examination was 11.3 min, with a SD of 2.31 min.

Therefore, to estimate the mean time for a dental examination within a margin of ± 1 min with a 95% confidence level, 20 estimates per role, were required. We analysed the data for all those who responded to the survey.

## Results

The demographic characteristics of the survey respondents are shown in Table 1. In total 41 dentists, 21 dental hygienist/therapists and 34 nurses completed the survey, the mean time qualified being 18.4, 13.8 and 17.8 years for these professional groups respectively. The proportion of patients treated via the NHS by dentists was 78.3%.

**Table 1:** Demographics of the respondents to the survey

SURVEY RESPONDENTS			
	Dentist (N = 41)	Therapist/Hygienist (N = 21)	Nurse (N = 34)
**Years qualified in Role (Years)** Mean (SD)	18.4 (10.7)	13.8 (9.9)	17.8 (11.1)
**Proportion of patients treated via the NHS (%) (**Mean SD)	78.3 (28.0)	65.8 (32.0)	71.8 (27.6)
**Where qualified** ^*^			
Qualified in the United Kingdom N (%)	37 (92)	21(100)	33 (97.1)
Qualified outside the United Kingdom N (%)	3 (7.3)	0(0)	0(0)

^*^May not sum to 100 due to missing data

The estimated time taken to complete clinical procedures by dentists for adult and child patients are illustrated in Tables 2 and 3.

**Table 2:** Mean time taken to undertake clinical procedures in adult patients as estimated by dentists

Clinical Procedure	ADULTS Average time (mins)	95% CI Low	95% CI High
Clinical note reading prior to patient	3.7	3.2	4.3
Preparing surgery for patient	3.8	3.1	4.5
Fluoride varnish application	3.9	2.6	5.2
Radiographs	4.6	3.8	5.3
Preventive advice	5.3	4.0	6.5
Plaque and bleeding scores	6.7	4.8	8.6
Smoking cessation	6.9	5.3	8.4
Clinical note writing	7.1	6.2	8.1
ACORN (Assessment of Clinical Oral Risks and Need)*	7.2	4.7	9.6
Prescription of medicines	7.9	6.4	9.3
Scale and polish	8.8	7.4	10.1
Six-point pocket chart	10.2	8.2	12.22
Examination routine	10.6	9.4	11.7
Examination acute conditions	11.4	9.6	13.2
Referral to specialist	13.1	11.2	15.1
Supra and subgingival scaling	20.8	15.7	25.8
Extraction	22.1	19.7	24.5
Fit of fixed restoration	22.7	20.5	245.0
Permanent filling or sealant restoration	23	20.9	25.1
Root surface debridement	30.1	25.1	35.0
Bridge prep resin retained	37.1	32.0	42.2
Veneer prep	41.6	37.4	45.8
Indirect inlay prep	43.4	37.5	49.3
Endodontic treatment permanent single rooted tooth	47.6	42.3	52.8
Endodontic treatment permanent multirooted tooth	77.4	69.0	86.0
Crown prep	49.9	44.5	55.2
Bridge prep conventional	62.8	56.0	69.7
Lower denture acrylic	68.8	58.7	78.8
Upper denture acrylic	69	59.1	79.0
Upper + lower denture acrylic	71.8	62.0	82.2
Lower denture metal	78.3	67.1	89.5
Upper denture metal	78.6	67.5	89.7
Upper + lower denture metal	81.8	69.5	94.0

*ACORN is a chairside oral health risk and need stratification tool used in primary dental care services in Wales [18]

**Table 3:** Mean time taken to undertake clinical procedures in child patients as estimated by dentists

Clinical Procedure	CHILDREN Average time (mins)	95% CI Low	95% CI High
Clinical note reading prior to patient	3.8	3.1	4.4
Preparing surgery for patient	3.4	2.5	4.4
Radiographs	5.5	4.5	6.5
Fluoride varnish application	4.4	3.5	5.8
Preventive advice	5.8	4.5	7.0
Clinical note writing	5.8	4.9	6.8
Plaque and bleeding scores	5.8	4.3	7.3
Scale and polish	5.3	4.1	6.5
Supra and subgingival scaling	8.9	6.6	11.3
Endodontic procedure on deciduous tooth	21.6	16.5	26.6
Definitive filling or sealant restoration of deciduous tooth	17.5	15.5	19.5
Extraction of deciduous tooth	17.6	15.0	20.1
Placement of preformed crown	21.8	15.4	28.2
Examination routine	7.6	6.6	8.6
Examination acute conditions	8.8	7.4	10.1
ACORN (Assessment of Clinical Oral Risks and Need)	6	4.8	7.2
Prescription of medicines	4.9	4.0	5.9
Referral to specialist	10.9	8.8	13.0
Endodontic treatment single rooted tooth	48.5	41.6	55.4
Endodontic treatment multirooted tooth	74.8	64.7	85.0
Extraction of permanent tooth	22.6	20.1	25.0

**Fig. 1:**
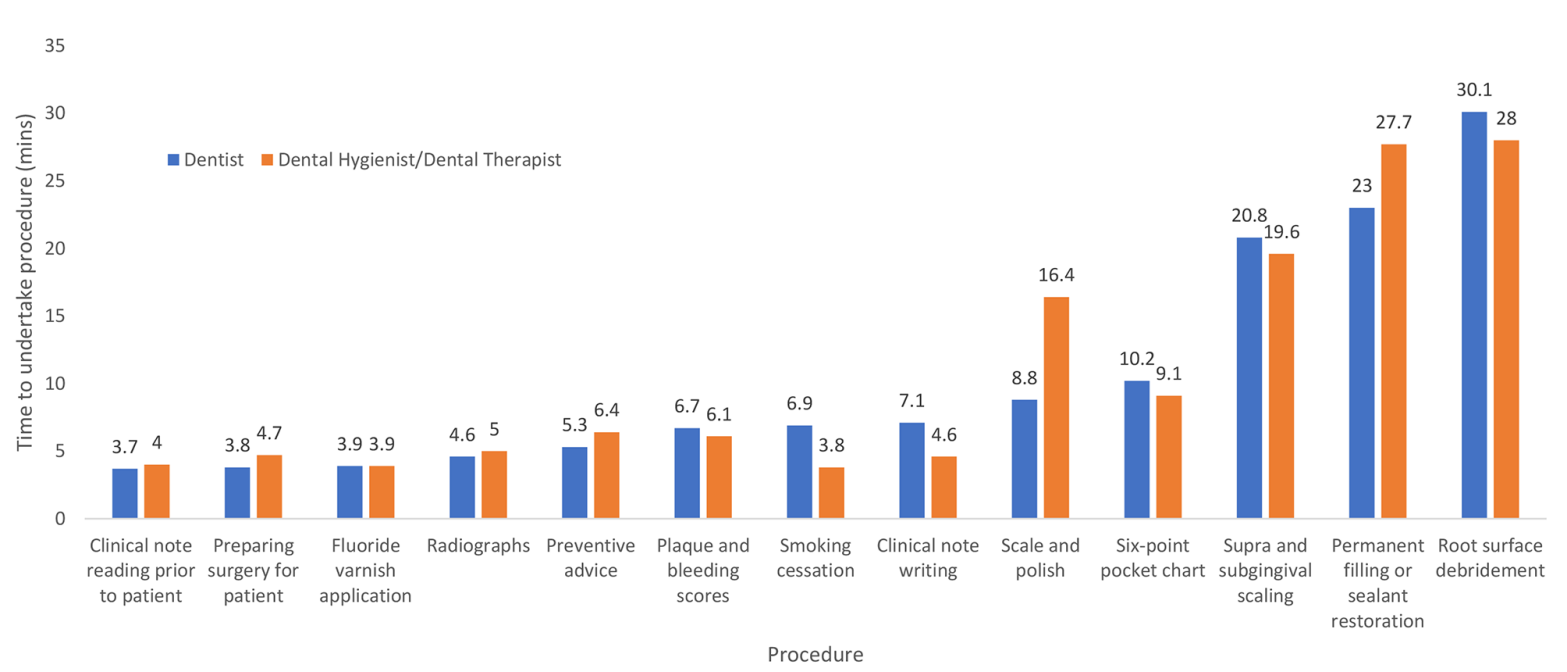
Mean time taken to undertake clinical procedures in adult patients as estimated by dentists and dental hygienists/dental therapists

**Fig. 2:**
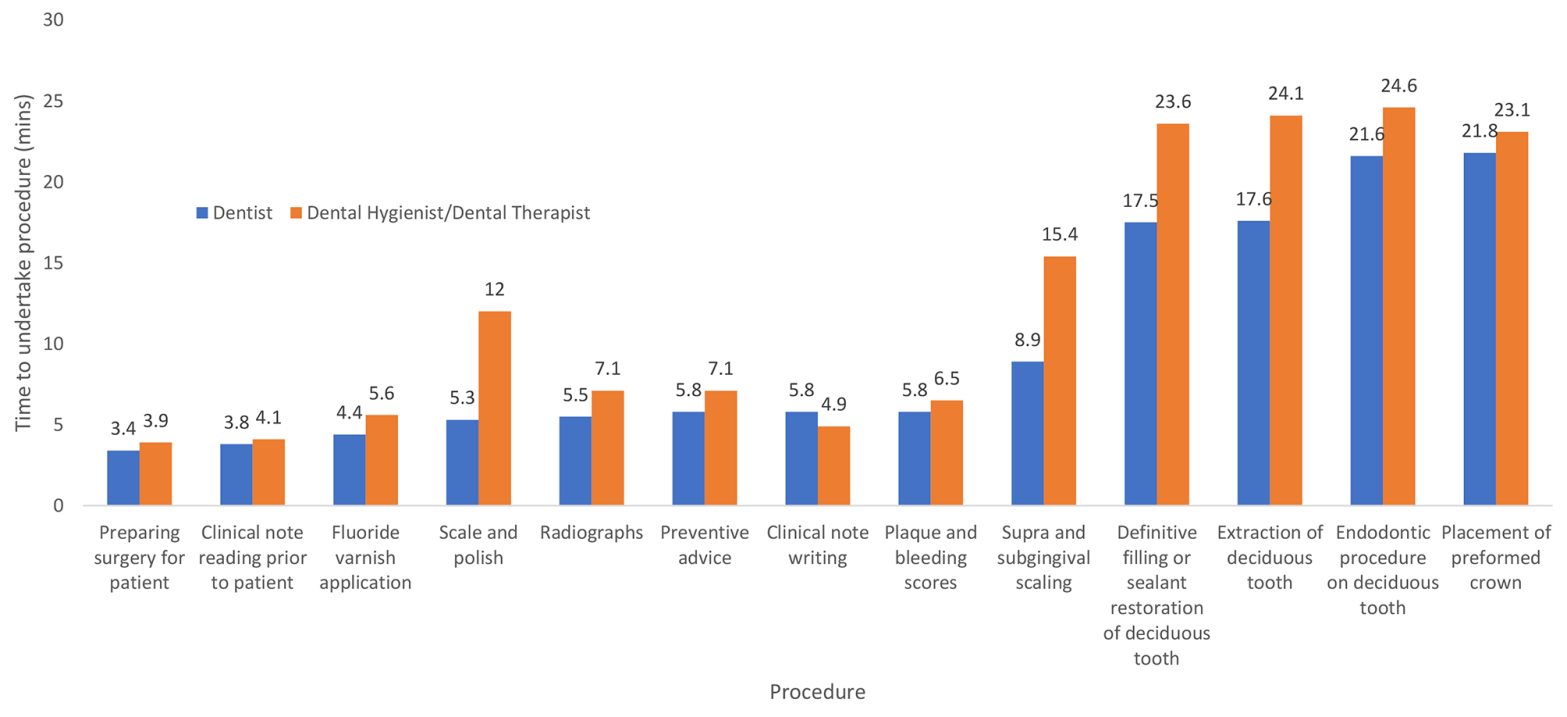
Mean time taken to undertake clinical procedures in child patients as estimated by dentists and dental hygienists/dental therapists

The estimated time taken to complete clinical procedures by dentists for adult and child patients compared with dental hygienists and therapists are illustrated in Figs. 1 and 2. Estimated times for adult patients were broadly similar and not statistically significantly different. The greatest difference occurred in the case of scale and polish where dentists said that this on average took 8.8 min compared with the dental hygienists and therapists whose estimate was almost double at 16.4 min. However, for six-point pocket charting (10.2 vs. 9.1), supra and subgingival scaling (20.8 vs. 19.6) and root surface debridement (30.1 vs. 28) dentists estimated marginally more time than the hygienists and therapists.

In the case of child treatments, overall the hygienists and therapists estimated that they spent a significantly greater period of time (*p* = 0.04) than did dentists undertaking similar procedures. For example they suggested more than twice the time required to undertake a scale and polish (12 min) compared with the dentists (5.3 min). Hygienists and therapists estimated a greater time to complete all other procedures except in the case of clinical note taking where they suggested this required 4.9 min compared with the mean of 5.8 min reported by dentists.

The time taken by dental nurses to undertake the various tasks that fall within their scope of practice are recorded in Supplementary Table A1.

A complete presentation of clinical timings as estimated by dentists, hygienists/therapists and dental nurses are presented in supplementary Tables A1-A3.

**Fig. 3:**
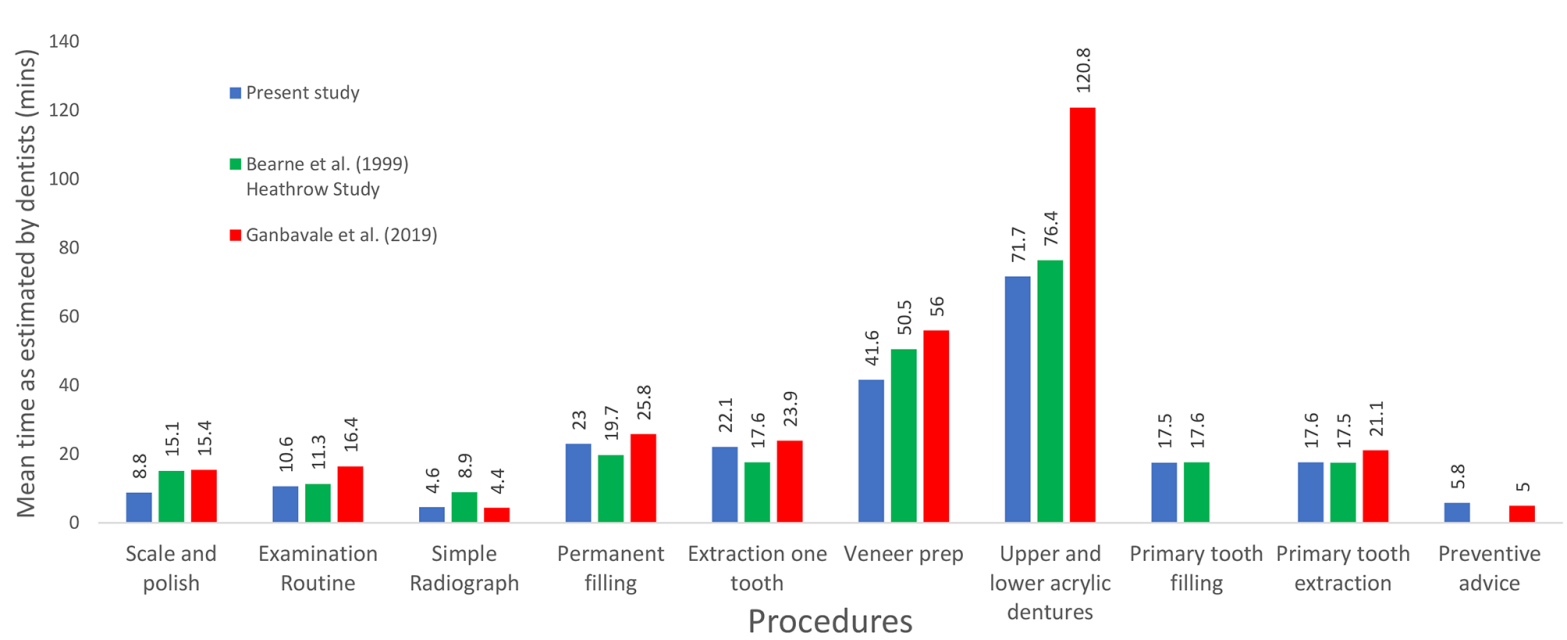
Comparison of time taken present study, Bearbe et al. 1999 (Heathrow study) and Ganbavale et al. (2019) studies as estimated by dentists

A complete comparison between the two previous studies [1, 2] and this one is not possible as different procedures were enquired after. For those procedures where direct comparison is possible the results are displayed in Fig. 3. From this it is apparent that the mean estimated time per procedure differed somewhat across the three studies, with the time taken to construct upper and lower acrylic dentures demonstrating the greatest variation.

## Discussion

This work provides contemporaneous estimated times taken to undertake routine dental procedures in NHS general dental practice, updating the often quoted “Heathrow Study” [1] and a more recent estimation by Ganbavale and colleagues [2]. The results here which focus on Wales, may not necessarily apply elsewhere in the United Kingdom.

The use of skill-mix as a means of delivering a dental care system with a greater emphasis on prevention has been described extensively [10–13, 15]. This work establishes differences in the perceived time taken by different members of the team to complete the same task. In the case of adult patients, the finding that there was no significant difference between the time taken by dentists and dental hygienists/therapists lends weight to the concept of delegating preventive and more simple restorative care to the dental hygienists and dental therapists. In contrast, why the dental hygienists and dental therapists took longer to undertake all procedures in children, except for clinical note writing, is unclear. These data suggest that in delegating care to dental hygienists and therapists there are unlikely to be savings in terms of time. They may also reflect that most dental therapist and hygienists in the UK are currently employed to provide periodontal treatment for adults rather than being able to provide full range of treatment within their scope of practice [17]. Similarly. comparing the cost of providing treatment items by dentists versus therapists and hygienists was not within the scope of this study. However, the difference in hourly rate/salary of dentists and dental therapist-hygienists may mean cost savings for employers provided that there are no barriers for using the full skill-set of therapists and hygienists.

There are limitations to the work that are worth mentioning. The times reported are estimates and the sample is purposive. The database to which invitations to participate were sent contained 3,869 individuals. This included all dental professionals registered with Health Education and Improvement Wales and included those working in the community and hospital dental services. It is therefore not possible to determine a response rate to the survey due to the lack of an accurate denominator. The sample size calculation suggested that a minimum of 20 participants per professional group was required. In our analysis we included all those who responded. It is accepted that there is likely an element of response bias in this approach. However, the methodology and sample size used in this study are similar to that of the original study on which the current timings for NHS dentistry have been based for more than two decades.

The estimates of how much time was spent on each item was when working under the NHS system should not be taken as the recommended time. It is also the case that time spent does not necessarily directly correlate to quality of care but is of course somewhat linked.

Of note is the relatively short times being reported to undertake root canal treatments. This likely reflects the pressures for undertaking these treatments in NHS practice. A limitation of how this was recorded means that it is not clear on whether this also is interpreted as including the time for undertaking the definitive restoration after completion of the endodontic treatment.

### Use of these data and future research

The data provided here will act as a useful resource for service planners and managers in developing and commissioning future dental services, particularly a service which envisages a greater degree of skill-mix and of preventive care. There is scope for further work to examine the differences in times estimated by different professional groups and to model how this might impact on a revised model of dental care delivery in the future.

## Conclusions

This work provides contemporary timings for commonly performed dental procedures in NHS general dental practice as undertaken by different members of the dental team. They may be helpful in designing a service to address current access issues. The reported time taken by dental hygienists and therapists suggest that they are similar to dentists when treating adults but take more time when treating children.

### Electronic supplementary material

Below is the link to the electronic supplementary material.

Supplementary Material 1

## Data Availability

Data utilised in this study are available by contacting the corresponding author.

## References

[1] Bearne A, Kravitz A (2000). The 1999 BDA Heathrow timings inquiry. Br Dent J.

[2] Ganbavale SG, Aukett JW, Gallagher JE (2019). Timings and skill mix in primary dental care: a pilot study. Br Dent J.

[3] Peres MA, Macpherson LMD, Weyant RJ, Daly B, Venturelli R, Mathur MR, Listl S, Celeste RK, Guarnizo-Herreno CC, Kearns C (2019). Oral Diseases: a global public health challenge. Lancet.

[4] Watt RG, Daly B, Allison P, Macpherson LMD, Venturelli R, Listl S, Weyant RJ, Mathur MR, Guarnizo-Herreno CC, Celeste RK (2020). The Lancet oral Health Series: implications for oral and Dental Research. J Dent Res.

[5] Watt RG, Steele JG, Treasure ET, White DA, Pitts NB, Murray JJ (2013). Adult Dental Health Survey 2009: implications of findings for clinical practice and oral health policy. Br Dent J.

[6] Wanyonyi KL, Radford DR, Harper PR, Gallagher JE (2015). Alternative scenarios: harnessing mid-level providers and evidence-based practice in primary dental care in England through operational research. Hum Resour Health.

[7] Dental Contract Reform https://www.nhsbsa.nhs.uk/dental-contractreform#:~:text=The%20aim%20of%20the%20dental%20contract%20reform%20programme,of%20clinically%20necessary%20treatments%20available%20on%20the%20NHS. Accessed 29 Jun 23.

[8] NHS England First stage of dental reform – 1. 2021 to 2022. https://www.england.nhs.uk/primary-care/dentistry/dental-commissioning/dental-contract-reform/ Accessed 29 Jun 23.

[9] Written Statement. : Dental Contract Reform 2022-23. https://www.gov.wales/written-statement-dental-contract-reform2022-23#:~:text=The%20oral%20health%20and%20dental%20services%20response%20to,underpins%20the%20delivery%20and%20payment%20for%20dental%20services. Accessed 29 Jun 23.

[10] Barnes E, Bullock A, Moons K, Cowpe J, Chestnutt IG, Allen M, Warren W (2020). A whole-team approach to optimising general dental practice teamwork: development of the skills optimisation self-evaluation Toolkit (SOSET). Br Dent J.

[11] Brocklehurst P, Macey R (2015). Skill-mix in preventive dental practice–will it help address need in the future?. BMC Oral Health.

[12] Brocklehurst P, Tickle M (2011). The policy context for skill mix in the National Health Service in the United Kingdom. Br Dent J.

[13] Cannell P (2016). Skill mix - a paradigm shift?. Br Dent J.

[14] Dyer TA, Owens J, Robinson PG (2014). The acceptability of care delegation in skill-mix: the salience of trust. Health Policy.

[15] Ward P (2006). The changing skill mix - experiences on the introduction of the dental therapist into general dental practice. Br Dent J.

[16] NHS Dental Services. [https://www.nhsbsa.nhs.uk/activity-payment-and-pension-services/dental-activity-processing]. Accessed 29 Jun 2023.

[17] Jones G, Evans C, Hunter L (2008). A survey of the workload of dental therapists/hygienist-therapists employed in primary care settings. Br Dent J.

[18] Cope AL, Bannister C, Karki A, Harper P, Allen M, Jones R, Peddle S, Walters B, Chestnutt IG (2022). The development and application of a chairside oral health risk and need stratification tool in general dental services. J Dent.

