# Supplementary material for: Time to complete contemporary dental procedures – estimates from a cross-sectional survey of the dental team
Source: BMC Oral Health. 2023 Nov 25;23:926. doi: 10.1186/s12903-023-03671-y (PMC10676590; doi:10.1186/s12903-023-03671-y)
Supplement: Supplementary file 1 — Supplementary Material 1 [file 12903_2023_3671_MOESM1_ESM.docx]

**Time to complete dental procedures: Supplementary Tables**

**SUPPLEMENTARY TABLES**

**Table A1: Estimated timings (in minutes) of common dental procedures performed by Dentists, Dental Hygienists/Therapists and Dental Nurses.**

| **Clinical Activity** | **Patient**  **Age Group** | **Dentists** | | **DH/DTs** | | **Nurses** | |
| --- | --- | --- | --- | --- | --- | --- | --- |
|  |  | Mean | Range | Mean | Range | Mean | Range |
| Clinical note reading prior to patient | Adult | 3.7 | 1.9-6.8 | 4.0 | 2.5-7.1 | 4.2 | 2.4-6.2 |
|  | Paediatric | 3.8 | 2.4-7 | 4.1 | 3-7.1 | 4.3 | 3.1-7.4 |
| Preparing.  surgery for patient | Adult | 3.8 | 2.2-6.6 | 4.7 | 3.7-7.4 | 6.0 | 4.3-8.8 |
|  | Paediatric | 3.4 | 2.4-6.1 | 3.9 | 3.2-6.8 | 5.2 | 4-8.3 |
| Radiographs | Adult | 4.6 | 3-6.7 | 5.0 | 3.4-7.5 | 4.6 | 3-6.2 |
|  | Paediatric | 5.5 | 4-9.3 | 7.1 | 6.2-10.7 | 6.0 | 5-10.1 |
| Fluoride varnish application | Adult | 3.9 | 2.3-5.1 | 3.9 | 2.4-5.1 | 5.2 | 3.6-7.6 |
|  | Paediatric | 4.4 | 2.6-6.1 | 5.6 | 3.8-8.2 | 6.6 | 5.4-9.9 |
| Preventive advice | Adult | 5.3 | 2.9-10.1 | 6.4 | 4.3-11.2 | 7.6 | 5.3-11.5 |
|  | Paediatric | 5.8 | 3.4-9.6 | 7.1 | 5.8-10.6 | 8.0 | 5.8-11.5 |
| Smoking Cessation | Adult | 6.9 | 4.9-10.7 | 3.8 | 3.3-5.9 | 5.4 | 4-8.1 |
| Clinical note writing | Adult | 7.1 | 4.2-15.2 | 4.6 | 3.4-9.2 | 6.9 | 5.1-11.2 |
|  | Paediatric | 5.8 | 3.8-12.8 | 4.9 | 3.8-8.8 | 6.2 | 4.7-9.7 |
| Cleaning surgery following patient | Adult |  |  | 5.8 | 4.6-8.8 | 7.1 | 6.9-10.6 |
|  | Paediatric |  |  | 5.3 | 5.4-9.2 | 6.7 | 4.5-9.6 |

**Table A2: Estimated timings (in minutes) of common dental procedures performed by Dentists and Dental Hygienists/Therapists.**

| **Clinical Activity** | **Patient**  **Age Group** | **Dentists** | | **DH/DTs** | |
| --- | --- | --- | --- | --- | --- |
|  |  | Mean | Range | Mean | Range |
| Six-point pocket chart | Adult | 10.2 | 7.4-14.8 | 9.1 | 6.1-12 |
| Plaque and bleeding scores | Adult | 6.7 | 4.2-8.9 | 6.1 | 3.6-6.8 |
|  | Paediatric | 5.8 | 3.8-8.5 | 6.5 | 4.5-8 |
| Scale and polish | Adult | 8.8 | 5.3-15.5 | 16.4 | 15-25.2 |
|  | Paediatric | 5.3 | 3.6-9.2 | 12.0 | 8.5-17.1 |
| Supra and subgingival scaling | Adult | 20.8 | 14.8-33.3 | 19.6 | 14.3-27.7 |
|  | Paediatric | 8.9 | 6-13.7 | 15.4 | 10.5-19.8 |
| Permanent filling or sealant restoration | Adult | 23.0 | 13.7-36.9 | 27.7 | 17.5-38.8 |
| Root surface debridement | Adult | 30.1 | 21.4-49.7 | 28.0 | 21.7-40.6 |
| Endodontic procedure on deciduous tooth | Paediatric | 21.6 | 13.5-28.4 | 24.6 | 21.2-30.2 |
| Definitive filling or sealant restoration of deciduous tooth | Paediatric | 17.5 | 11.4-30.3 | 23.6 | 16.5-29.6 |
| Extraction of deciduous tooth | Paediatric | 17.6 | 11.3-27.2 | 24.1 | 17.1-32.5 |
| Placement of preformed crown | Paediatric | 21.8 | 16.5-29.5 | 23.1 | 19.7-29.3 |

**Table A3: Estimated timings (in minutes) of common dental procedures performed by Dentists.**

| **Clinical Activity** | **Patient Age Group** | **Dentists** | |
| --- | --- | --- | --- |
|  |  | Mean | Range |
| Examination routine | Adult | 10.6 | 6.3-19.4 |
|  | Paediatric | 7.6 | 4.5-13.8 |
| Examination acute conditions | Adult | 11.4 | 7.1-19.7 |
|  | Paediatric | 8.8 | 5.4-16.3 |
| ACORN | Adult | 7.2 | 5.6-11.4 |
|  | Paediatric | 6.0 | 4.5-9.8 |
| Endodontic treatment permanent single rooted tooth | Adult | 47.6 | 33.4-62.9 |
| Endodontic treatment permanent multi rooted tooth | Adult | 77.4 | 56.5-109.6 |
| Extraction | Adult | 22.1 | 13.4-44.9 |
| Upper denture acrylic | Adult | 69.0 | 52.8-93.5 |
| Lower denture acrylic | Adult | 68.8 | 52.5-93.5 |
| Upper+lower denture acrylic | Adult | 71.7 | 56.1-97.8 |
| Upper denture metal | Adult | 78.6 | 64-101.3 |
| Lower denture metal | Adult | 78.3 | 63.7-101.3 |
| Upper+lower denture metal | Adult | 81.8 | 66.7-106.9 |
| Crown prep | Adult | 49.9 | 37.7-66.3 |
| Veneer prep | Adult | 41.6 | 32.1-53.9 |
| Indirect inlay prep | Adult | 43.4 | 33.4-59.7 |
| Bridge prep conventional | Adult | 62.8 | 49.6-88.3 |
| Bridge prep resin retained | Adult | 37.1 | 28.6-49.9 |
| Fit of fixed restoration | Adult | 22.7 | 16.2-35.6 |
| Prescription of medicines | Adult | 7.9 | 5.4-12.8 |
|  | Paediatric | 4.9 | 3.5-7.5 |
| Referral to specialist | Adult | 13.1 | 9.9-21.9 |
|  | Paediatric | 10.9 | 7.9-17.5 |
| Inhalation sedation | Adult | 25.0 | 15-37.5 |
|  | Paediatric | 25.0 | 20-45 |
| IV sedation | Adult | 45.0 | 30-120 |
|  | Paediatric | 40.0 | 30-120 |
| Endodontic treatment single rooted tooth | Paediatric | 48.5 | 35-67.2 |
| Endodontic treatment multirooted tooth | Paediatric | 74.8 | 60.2-133 |
| Extraction of permanent tooth | Paediatric | 22.6 | 15.3-36.8 |
